# Extracellular Vesicles in Mental Disorders: A State-of-art Review

**DOI:** 10.7150/ijbs.79666

**Published:** 2023-02-05

**Authors:** Lingzhuo Kong, Danhua Zhang, Shu Huang, Jianbo Lai, Lin Lu, Jing Zhang, Shaohua Hu

**Affiliations:** 1 Department of Psychiatry, the First Affiliated Hospital, Zhejiang University School of Medicine, Hangzhou 310003, China.; 2 The Key Laboratory of Mental Disorder's Management in Zhejiang Province, Hangzhou 310003, China.; 3 Brain Research Institute of Zhejiang University, Hangzhou 310003, China.; 4 Zhejiang Engineering Center for Mathematical Mental Health, Hangzhou 310003, China.; 5 Department of Neurobiology, NHC and CAMS Key Laboratory of Medical Neurobiology, School of Brain Science and Brian Medicine, and MOE Frontier Science Center for Brain Science and Brain-machine Integration, Zhejiang University School of Medicine, Hangzhou 310003, China.; 6 Peking University Sixth Hospital, Peking University Institute of Mental Health, NHC Key Laboratory of Mental Health (Peking University), National Clinical Research Center for Mental Disorders (Peking University Sixth Hospital), Chinese Academy of Medical Sciences Research Unit (No.2018RU006), Peking University, Beijing, China.; 7 Peking-Tsinghua Center for Life Sciences and PKU-IDG/McGovern Institute for Brain Research, Peking University, Beijing, China.; 8 Department of Pathology, First Affiliated Hospital, Zhejiang University School of Medicine, Zhejiang, China.; 9 National Health and Disease Human Brain Tissue Resource Center, Zhejiang University, Zhejiang, China.

**Keywords:** Extracellular vesicle, non-coding RNA, mental disorder, biomarker

## Abstract

Extracellular vesicles (EVs) are nanoscale particles with various physiological functions including mediating cellular communication in the central nervous system (CNS), which indicates a linkage between these particles and mental disorders such as schizophrenia, bipolar disorder, major depressive disorder, etc. To date, known characteristics of mental disorders are mainly neuroinflammation and dysfunctions of homeostasis in the CNS, and EVs are proven to be able to regulate these pathological processes. In addition, studies have found that some cargo of EVs, especially miRNAs, were significantly up- or down-regulated in patients with mental disorders. For many years, interest has been generated in exploring new diagnostic and therapeutic methods for mental disorders, but scale assessment and routine drug intervention are still the first-line applications so far. Therefore, underlying the downstream functions of EVs and their cargo may help uncover the pathogenetic mechanisms of mental disorders as well as provide novel biomarkers and therapeutic candidates. This review aims to address the connection between EVs and mental disorders, and discuss the current strategies that focus on EVs-related psychiatric detection and therapy.

## Introduction

**Fig 1:**
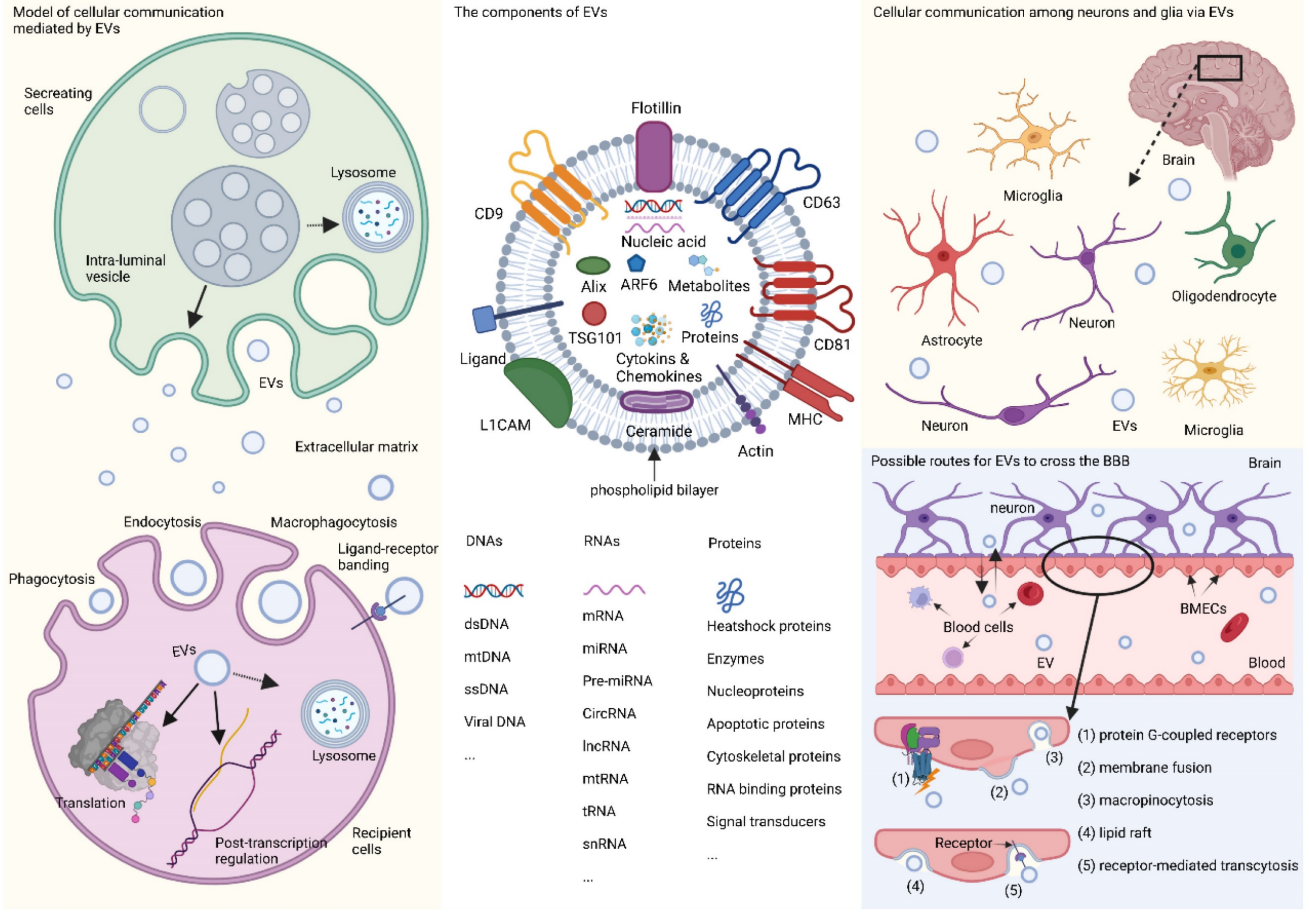
** EVs and their participation in cellular communication in the brain.** EVs originate primordially as multivesicular bodies from intraluminal vesicles and are generated by inward budding, encapsulating nucleic acids, proteins, metabolites, and other components, some of which are recognized as detecting markers, such as tetraspanins, Alix, TSG101, ARF6, and L1CAM. Fusion of multivesicular bodies membrane with cell membrane facilitates the release of EVs, while fusion with lysosomal membrane leads to the degradation of components. When entering the recipient cells via macropinocytosis, phagocytosis, and endocytosis with or without ligand-receptor binding, EVs will end up in the lysosome, or participate in the post-transcription regulation, or translate their capsuled RNAs into functional proteins. In the brain, EVs are released by neurons and glia and mediate cellular communication between different neurons, or between glia and neurons. Furthermore, peripheral EVs can cross the BBB via five possible pathways theoretically, namely, association with a protein G-coupled receptor, surface adhesion and membrane fusion, macropinocytosis, lipid raft, and receptor-mediated transcytosis. EVs: extracellular vesicles; L1CAM: L1 cell adhesion molecule; TSG101: tumor susceptibility gene 101; CNS: central nervous system; BBB: blood-brain barrier; Alix: apoptosis-linked gene 2-interacting protein X; ARF6: adenosine diphosphate ribosylation factor 6; MHC: major histocompatibility complex

Extracellular vesicles (EVs) are minute phospholipid bilayer particles with diameters ranging from ~30 nm to 10 μm, and are typically classified into four sub-categories, exosomes (~30 nm to 150 nm), ectosomes (~100 nm to 1000 nm), apoptotic bodies (~1000 to 5000 nm), and oncosomes (~1 μm to 10 μm).^1^, ^2^ The heterogeneity and role of EVs are predominantly determined by their cargo, such as nucleic acids, proteins, lipids, cytokines, chemokines, the end-stage neurotoxic and pathogenic metabolic products.^3^-^5^ Among these contents, RNAs have drawn special attention. RNAs encapsulated in EVs mainly include non-coding RNAs such as mRNAs, long non-coding RNAs (lncRNAs), circular RNAs, and the widely-studied microRNAs (miRNAs or miR),^3^ which have been proposed as emerging diagnostic or therapeutic candidates for many neuropsychiatric diseases.^6^, ^7^ When transferred from cell to cell, RNAs in EVs can mediate the orientation, translation, and stability of mRNA in the target cells by combining with trans-acting factors, such as RNA-binding proteins, thus controlling cell development and differentiation.^8^ Meanwhile, the pathophysiological characteristics of EV-derived lncRNAs, circular RNAs, and mRNA have also been gradually revealed over the years.^9^-^11^ The identification of EVs is based on molecular biomarkers such as tetraspanins (e.g., CD9, CD63, CD81), syntenin-1, apoptosis-linked gene 2-interacting protein X (Alix), TSG101, neuronal origin marker L1 cell adhesion molecule (L1CAM), and adenosine diphosphate ribosylation factor 6 (ARF6).^12^ Some of these markers can even indicate the different origination of EVs. For example, L1CAM is commonly recognized as the label of EVs derived from neurons in the central nervous system (CNS),^4^, ^5^, ^7^ and other biomarkers such as glutamate aspartate transporter and transmembrane protein 119are indications of glia-derived EVs.^13^ In recent years, studies on the classification, biological function, and protocols for isolation and detection of EVs are under rapid growth (**Fig. 1**).

In the CNS, EVs can be secreted by different cells and act as message carriers during neighboring and distal cellular communication between neurons, glia, and other types of cells,^14^-^23^ thereby mediating a cascade of downstream reactions. EVs are capable of crossing the blood-brain barrier (BBB) via five regular routes (shown in **Fig. 1**) 16, 24 and can be detected in various body fluids, and have been proposed as possible biomarkers neurodegenerative disease and CNS tumors,^25^-^27^ which open a window for us explore the physiological and morbid status of the brain non-invasively.^28^

## EVs and mental disorders

**Fig 2:**
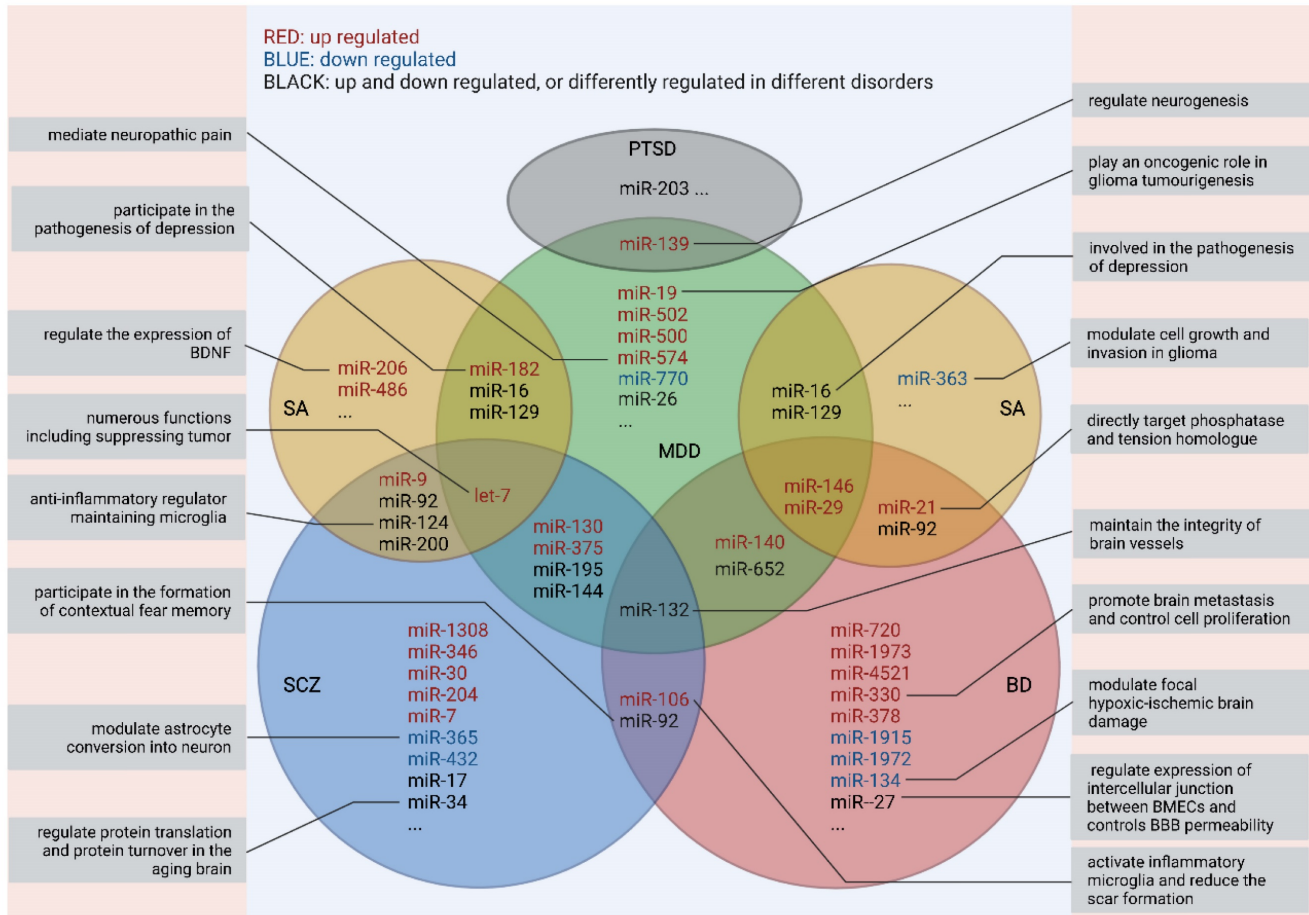
Overlaps in peripheral miRNAs across different studies and their functions* *Only RNA families were identified, but not limited to specific homologous miRNAs as some of the research findings have not been replicated yet. All the miRNAs presented above are detected from serum/plasma. MDD: major depressive disorder; BD: bipolar disorder; SCZ: schizophrenia; SA: substance abuse; PTSD: post-traumatic stress disorder

Up till now, the pathological mechanisms of most mental disorders remain largely unclear, thus hindering the innovation of strategies for diagnosis and treatment.^29^ Inspiringly, EVs are a mirror that can reflect the real-time status of the brain and specific clinical symptoms like non-suicidal self-injury 30 and cognitive impairment.^31^ Ongoing experimental and technological advances have been yielding remarkable findings on the physiological effects and heterogeneity of EVs and their cargoes,^3^ and have shown the potential to harness EVs for prevention, prediction, precise diagnosis, and treatment of neuropsychiatric disorders. For example, insulin receptor substrate -1 (IRS-1), a protein associated with insulin resistance, was increased in neuron-delivered EVs from patients with MDD, thus indicating the relationship between insulin resistance in the CNS and clinical symptoms such as depressive feelings, suicide and anhedonia.^32^ However, among various components in EVs, miRNA has been adequately explored and seems to be the most promising biomarker. Despite conflicting findings, accumulating studies of schizophrenia, major depressive disorder (MDD), bipolar disorder (BD), substance abuse (SA), and post-traumatic stress disorder (PTSD) have yielded early evidence on dysregulation in EV-derived miRNAs (**Fig. 2**).

Notably, none of those dysregulated miRNAs showed specificity for a given disorder or has been repeatedly verified by different studies. Thus, more endeavor is needed to make further explorations of the EV-related RNA candidates before their clinical applications.

### The neuropathological underpinnings

#### EVs implicated in the pathophysiological processes in the CNS

Mental disorders are not simply the results of brain dysfunction, they have pathophysiological underpinnings. EV-mediated myelin impairment of neurological diseases^33^ are also implicated in different mental disorders.^6^, ^34^-^41^ EVs participate in cellular communication between neurons and glia, which is known as “neuroimmune interactions”, and it is the basis of the pathophysiological processes including decreased synaptic activity, elimination of waste, and maintenance of myelin integrity.^19^, ^42^ The most critical cell population participating in the neuroimmune interactions is microglia. Generally, EVs are important vehicles of microglia-related bioactive molecules which are delivered towards other CNS-resident cells, while the phenotype and physiological status of microglia could also be regulated by EVs from other cells.^43^ EVs secreted by activated microglia are enriched in selective pathogenetic miRNAs and other bioactive factors that are entangled in intercellular interactions such as complement activation and neuroinflammation, which may disrupt innate immune signaling as well as neurotropism and synaptic signaling progress through regulating expression of neuron-specific phosphoproteins.^44^-^46^ In addition, the differentiation and myelin deposition of oligodendrocytes that form a highly specialized functional entity could be influenced by microglia-derived EVs, which is critical for the remyelination process in neuroinflammatory diseases.^47^

EVs derived from astrocytes participate in neuroimmune interactions as well as the central-to-peripheral immune communication.^48^ On the one hand, the astrocytes-derived pro-inflammatory EVs can be taken by neurons and lead to neuronal damage.^17^, ^48^ Meanwhile, those EVs can mediate microglia activation, migration, and phagocytic capacity, accounting for neuron inflammation.^17^, ^49^ On the other hand, in the physiological state, astrocyte-derived EVs prevent inflammation, facilitate tissue repair, and enhance physiological adaption to the inflammatory micro-environment during the acute morbid stage.^50^-^52^ Furthermore, EVs could be produced by both CNS-resident and CNS-infiltrating leukocytes under the circumstance of neuroinflammation,^42^ regulating the activity of neuron and glia cells in the CNS.

Internalized by neurons in response to stimuli, EVs derived from oligodendrocytes have neuroprotective properties and are indispensable for the homeostasis of the CNS micro-environment.^53^ Oligodendrocyte-specific genetically defective mice secreted fewer EVs-delivered proteins, resulting in a deficit of neuronal long-term stability.^54^ In addition, many myelin proteins relevant to autoimmune encephalomyelitis were found in EVs cultured *in vitro*,^55^ thus shedding light on drug delivery for CNS neuroimmune diseases such as multiple sclerosis.^56^ Recently, myelin deficits have been documented as new neuroimaging characteristics in patients with recurrent MDD.^33^ but the role of EVs in myelin deficits is still unclear.

#### EVs increase the permeability of the BBB

Leakage of the BBB, which is related to abnormalities in glutamatergic transmission and neuroinflammation,^57^ has been observed in neurodegenerative diseases and some types of mental disorders.^24^, ^58^-^60^ Proteomic studies also revealed that several neurodegenerative diseases biomarkers such as amyloid precursor protein and prion protein were enriched in neuron-derived or glia-derived EVs in human cerebrospinal fluid (CSF),^61^ aligning with cognitive decline in several mental disorders including schizophrenia,^62^ later-life MDD,^63^ autism spectrum disorder (ASD),^64^ sleep disorder (SD),^65^ and PTSD.[65]Therefore, EVs might be involved in the dissemination of the pathological changes from brain to other tissues.

Notably, EVs may participate in regulating the permeability of the BBB. According to a study using a zebrafish model, EVs containing miR-132 derived from the brain could target directly at the tight junction between brain microvascular endothelial cells (BMECs), resulting in increasing BBB permeability and microhemorrhage risk in the CNS.^66^

Nevertheless, we should be cautious with the impact of EVs on the BBB. It has been demonstrated that brain-derived EVs could either protect BMECs from the hypoxia damage^67^ and maintain the integrity of brain vessels,^66^ or disrupt the homeostatic permeability of the BBB. Although relevant research is limited,^68^ the increased BBB permeability acts as a bridge combining the dysregulation of EVs with the dysfunction of brain, indicating the potential of targeting EVs for the treatment of mental disorders.

## EVs and major mental disorders

### Schizophrenia

The clinical manifestations of schizophrenia, a prevalent mental disorder with complex etiology, are characterized by high heritability, early-onset, and debilitating course.^69^,^70^ The pathological mechanisms of schizophrenia are still indistinct, but the linkage between schizophrenia and decreased dopamine in the prefrontal cortex, excessive dopaminergic activity in the mesolimbic tract, decreased mesocortical dopaminegic neurons and gamma-aminobutyric acid (GABA)-ergic inhibitory activities, has been widely recognized.^71^ Increasing evidence also indicates that epigenetic modification may be associated with the pathogenesis of schizophrenia,^72^ while EV and its encapsulated miRNAs may fitly act as epigenetic regulators.^73^

Neuron- or glia-derived EVs may contribute to the onset of schizophrenia by transporting toxic (or misfolded) proteins and neurotransmitters, resulting in clinical symptoms like cognitive deficits. 25 perturbed metabolites in EVs related to metabolism of glycerophospholipid, phenylalanine, tyrosine, or tryptophan have been found to be linked to schizophrenia.^74^ In detail, proteins or small molecules such as amyloid-beta 42 (Aβ42) 62 and glial fibrillary acidic protein was elevated, while α-II-spectrin^75^, subunits 1 and 6 of NADH-ubiquinone oxidoreductase and subunit 10 of cytochrome b-c1 oxidase were lower in patients with schizophrenia, which provides evidence on the astrocyte-related neuroinflammatory underpinnings of the association between the dysregulated protein expression and increased reactive oxygen species.^76^ Other research discovered dysregulation of miRNAs in EVs from postmortem brain tissues, CSF and peripheral blood in patients with schizophrenia or other mental disorders with psychosis. Moreover, elevated phosphatidylserine-positive EVs were detected in the CSF of schizophrenia patients, which might be a signature of enhanced membrane shedding in the CNS.^77^ Studies on blood EV-related miRNAs indicate that specific miRNAs might contribute to the pathogenesis of schizophrenia including abnormalities in protein glycosylation, neurotransmitter biosynthesis, and dendrite development, and eventually reshape the structural and functional phenotype of the brain.^78^, ^79^ For example, miR-132 in EVs increased in MDD and BD while it decreased in SCZ, indicating the different pathological mechanism between mood disorders and SCZ, such as the *CLOCK* gene, a target gene of miR-132.^68^ Besides, miR-132 has a broad impact on the nervous system. It not only regulates the differentiation, maturation and functioning of neuron, but also widely participates in axon growth and neural plasticity, and thus preventing hippocampal neurogenesis and relevant memory deficits.^80^, ^81^ Other miRNAs are also important. miR-106b-5p, an inflammation- and tumor-associated miRNA, was reported in both schizophrenia and BD studies, while miR-195-5p, miR-181b, miR-144-5p, miR-130b, and let-7g were documented in both schizophrenia and MDD subjects, indicating the pathological mechanisms of dysregulation of AKT pathway, cytokine production, macrophage polarization, energy metabolism in those disorders,^68^ thus providing a plausible explanation for the overlapping symptom spectrum across different mental disorders.

However, in-depth explorations on the role of EV-related miRNAs in accessing clinical symptoms and treatment outcomes of schizophrenia are limited. Patients with early-onset schizophrenia showed up-regulated exosomal miR-137 and decreased cytochrome c oxidase complex IV COX6A2, which is involved in the psychological functions of mitochondria.^82^ This suggests that alternations of miR-137/COX6A2 EVs in plasma may represent a proxy marker of cortical microcircuit impairment, a pathological feature of schizophrenia.^82^ Besides, the level of miR-223, an EV-related miRNA targeting glutamate receptors, was increased in the orbitofrontal cortex of patients with schizophrenia.^83^ Following treatment with olanzapine or haloperidol, the expression of miR-223 was down-regulated while the expression of neuronal Grin2b was increased, thus revealing that miR-223 might be a promising treatment target of schizophrenia. Therefore, further investigation on the cargo of EVs may offer insights into the etiology of schizophrenia and new treatment strategies.

In terms of treatment, several studies revealed that EVs could alleviate the toxicity of glutamate overload in specific regions of the brain. In phencyclidine-induced schizophrenia mice model, mesenchymal stem cell-derived EVs can migrate to the prefrontal cortex by intranasal delivery, a brain area that is critically involved in the neuropathology of schizophrenia. Following EVs treatment, social interaction and disruption due to pre-pulse inhibition were significantly improved, parvalbumin-positive GABAergic interneurons were preserved, and the level of glutamate in the CSF was decreased,^84^ thus indicating a potentially novel treatment strategy for schizophrenia.

### Bipolar disorder

The pathophysiology of BD is complex. Neuroinflammation, disturbed neurogenesis and neuroplasticity are found to underline the recurrent depressive or manic/hypomanic attacks of BD.^85^ Interestingly, EVs are closely bound up with the pathological mechanisms of BD, and act as emerging biomarkers for detecting BD clinically. Omics research has provided further evidence. Dozens of dysregulated miRNAs, among which some were involved in netrin-mediated axon guidance as well as signaling pathways of the endothelium, serotonin, and androgen, have been detected in BD cases, and a few miRNAs showed phase-specific alterations in BD patients.^86^, ^87^

Alternations in EVs detected from BD patients, as well as their cargoes, are potential biomarkers for identifying phases of BD. Metabolites produced by galactose or amino sugar metabolism like xylitol were decreased in serum EVs from patients with BD, and a random forest classifier constructed by 15 exosomal metabolites showed excellent performance in diagnosis and differential diagnosis for BD.^88^ Astrocyte-derived EVs might transfer the stress signals such as cytokines and corticosteroids from peripheral blood, thereby causing deficits in neurogenesis.^89^ The encapsulated miRNAs in EVs can regulate both synaptic plasticity and brain development. For example, miR-134 was associated with the development of dendritic spines and synapses, and was a biomarker for monitoring mania episodes in BD.^90^ Moreover, miR-128 and miR-378 were also found to be linked to neurite outgrowth and neurogenesis in BD cases.^91^

Notably, the change of miR-142-3p was inconsistent in different studies, 86, 87 and in patients with type II BD, an elevated level of miR-142-3p was more likely to be detected in EVs from serum.^92^, ^93^ miR-29c, which played a role in neural development and signal transduction in the CNS, was up-regulated in BD cases, and was proposed as a target of lithium treatment.^94^ miR-29a, which was also up-regulated in BD patients, was critically involved in the functional and structural neurotoxic injuries.^95^ miR-149 can inhibit glial proliferation.^96^ It has been observed that miR-149 in EVs was altered in the anterior cingulate cortex (ACC) of BD patients,^97^ and an increased miR-149 in EVs led to a reduced number of glial cell in the ACC of familial MDD and BD patients.^98^, ^99^

Besides, recent research have suggested that the gut-brain axis (GBA) may be implicated in the pathogenesis of BD,^100^, ^101^ with increasing evidence revealing the disturbance of the intestinal microbiota and disruption of the bidirectional interaction between the brain and the gut microbes in BD individuals.^102^-^104^ A significant discrepancy was found both in the serum and intestinal biomarkers of microbiome between BD cases and healthy controls.^105^ A recently published review comprehensively discussed the involvement of brain- and gut-derived EV miRNAs in the GBA-related pathways (i.e., miR-375-3p, and miR-294-5p),^106^-^108^ indicating a novel regulatory strategy targeting the GBA via EVs modulation.^109^

EVs also show a great potential in liquid biopsy in clinical practice. The levels of molecules in neuron origin EVs such as p-NF-κB and p-FADD were associated with anhedonia and treatment outcome of infliximab.^110^ Moreover, EVs can reflect the cerebral status of insulin resistance, which are related to the cognitive function as well as the structure of the brain.^111^ For example, the neuronal-enriched EV indicated that biomarkers of insulin signaling were associated with cognitive function and brain structures independently, and following infliximab treatment, improvement in anhedonic symptoms and decrease in inflammatory molecules was linked to an enhanced insulin signaling via neuron-derived EVs.^110^, ^112^ Therefore, EVs provide a new insight into the pathological mechanisms of BD, which may facilitate the diagnosis and treatment in the future.

### Major depressive disorder

Current knowledge on the pathophysiology of MDD includes alterations in neural and glial activity, hypo-connectivity of frontoparietal network, and overactivation in the hypothalamic-pituitary-adrenal axis, which contribute to disturbance in neruotransmitter transmission, as well as structural and functional abnormalities in the brain.^113^, ^114^

Findings of pre-clinical studies provide preliminary evidence that EVs are involved in the pathophysiological progression of MDD, of which the most well-known mechanisms are neuroinflammation and neuroplasticity. Activation of microglial cells in different regions of the CNS was observed in MDD patients, accompanied with a decrease in neurogenesis and an increase in glutamate toxicity.^115^-^117^ EVs released by microglia encapsulate inflammatory cytokines and miRNAs that are crucial in neurogenesis, neurotransmission, and ion channel regulation.^118^, ^119^ It has been reported that EV-related miR-9-5p promoted microglial M1 polarization and over-released cytokines, such as IL-1β, IL-6, and TNF-α, thus exacerbating nerve injury.^120^ Fan et al.^121^ observed that the overexpression of microglia-enriched miRNAs, such as miR-146a-5p in the hippocampal dentate gyrus of mice, can suppress neurogenesis and spontaneous discharge of excitatory neurons, and act on the contrary when it was down-regulated. Furthermore, miR-207 in the EVs derived from natural killer cells alleviated depressive symptoms mice by targeting Tril-NF-κB pathway in astrocytes.^122^ The miR-26a and miR-186-5p were dysregulated in the hippocampus from animal models of MDD, which may be caused by the down-regulation of SERPINF1 encapsulated in EVs derived from the bone marrow mesenchymal cells and serum.^123^, ^124^

Clinical studies have also demonstrated the potential of cargoes in EVs for assisting the diagnosis of MDD. Molecules in EVs, such as pro-BDNF, miR-130b, miR-361-5p, miR-140-3p, miR-574-3p, miR-139-5p and miR-335-5p, were up-regulated in MDD cases, while BDNF, miR-34c-5p and miR-770-5p, miR-1292-3p, let-7b and let-7c were down-regulated.^125^-^127^ Notably, higher miR-9-5p level was detected in serum- EVs of MDD patients, suggesting the connection between microglia-mediated neuroinflammation and MDD pathophysiology.^120^ Additionally, the concentration of mitochondrial proteins and metabolic proteins such as insulin receptor substrate-1 in L1CAM enriched EVs, was increased in MDD patients, indicating dysregulation of neuronal mitochondrial activity and metabolic progress in CNS.^32^, ^128^ Those findings suggest that changes of EVs and their cargoes can be used as disease markers for monitoring the pathological changes of MDD.

Intriguingly, changes in behavioral phenotypes were also linked to the dysregulated level of EVs and their cargoes, showing the potential application of EVs in accessing the susceptibility, severity and symptoms of MDD. It was found that miRNAs in EVs could distinguish the susceptibility to chronic social defeat stress-induced social avoidance phenotype in mice, which may be due to the production of pro-inflammatory cytokines after injection.^129^ Furthermore, downregulation of miR-146a-5p and Exosomal sigma-1 receptor was proven to ameliorate depressive-like behaviors in model mice.^121^, ^130^ As for clinical findings, some molecules in EVs are associated with specific symptoms. For example, ISR-1 in neuron-derived EVs was related to the feeling of guilt, suicidality and anhedonia, while a higher level of pSer-IRS-1 was correlated to an aggravated severity of depression in females. 32 Furthermore, levels of miR-21-5p, miR-30d-5p, and miR-486-5p changed significantly in peripherally extracted neuron-derived EVs from patients who had favorable responses to antidepressant treatment, and the molecular targets of these miRNAs were also altered in the brain of depressed individuals,^131^ suggesting the application of EVs in monitoring the treatment outcome of antidepressive treatment in the future.

In terms of the utility of EVs in MDD treatment, miR-146a-5p enriched EVs, RVG-circDYM engineered EVs, and miR-207 containing EVs showed the ability to alleviate depression-like behavior in mice. 121, 122, 132 However, there is still a long way to verify the accuracy and sensitivity of these biomarkers before clinical application.

## EVs and other mental disorders

Relevant research has also indicated the critical role of EVs in other mental disorders including ASD, SA, SD, and PTSD. The latest and representative pre- and clinical studies are summarized in **Table 1**. It is noteworthy that overlaps of susceptibility between these mental disorders and aforementioned disorders including schizophrenia, MDD, and BD have been reported previously,^133^-^140^ which could probably be medicated by the dysregulation of the same EVs or their cargoes.

### Autism spectrum disorder

ASD is an early onset (usually in the first three years after birth) mental disorder which is characterized by impaired social communication as well as repetitive and restricted patterns of behaviors, interests, or activities.^141^ Several molecules targeting on key synaptic genes were found to be related to ASD through pathological process including neuronal inflammation, microglial activation and abnormal growth.^142^, ^143^ Emerging evidence indicates that dysfunction of synaptic and BBB-related gene expression regulated by miRNAs (i.e., miR-146a, miR-221, miR-654-5p, and miR-656) and other components such as mitochondrial DNA, lncRNA and mRNA derived from EVs originating in the CNS may contribute to the abnormalities in the growth of microglial and cause neuroinflammation, which was considered as a pathological landmark of ASD.^144^-^147^ Notably, up-regulated expression of neuroinflammatory genes may also influence BBB integrity in ASD patients.^142^ EVs are capable of mediating the increased permeability of the BBB, which further aggravates neuroinflammation. Therefore, EVs may be involved in the pathological mechanism of ASD via impairing the BBB function and inducing CNS neuroinflammation.

Interestingly, stem cell-derived EVs have the therapeutic potential for ASD since EVs can be directly delivered to the prefrontal cortex after nasal administration.^148^, ^149^ Intranasal administration of mesenchymal stem cells derived EVs was found to relieve autistic-like behaviors in two mice models.^141^, ^150^ Bone marrow-extracted mesenchymal stem cell (MSC)-derived exosomes showed the capacity to inhibit the release of pro-inflammatory molecules such as TNF-α and IL-1β,^151^ which might in turn reduces the problem behaviors, such as irritability and decreased socialization in ASD patients.^143^ However, clinical studies of EVs in ASD is still insufficient and calls for more endeavor.

### Substance abuse

SA is a multifactorial syndrome resulting from a complex interplay between the reward circuitry. Research evidence revealed that SA might promote the release of endogenous EVs with changed cargoes, suggesting that exposure to addictive agents may disrupt the EV-mediated signal transduction.^152^ A recent review comprehensively overviewed the current knowledge on EVs and their relationship with SA,^153^ and concluded that cargo of EVs such as toll-like receptor 4, miR-146a, miR-182,^154^ miR-124,^155^ miR-21, miR-126,^156^ miR-9-3p, 157 miR-21-3p,^158^ miR-15b, miR-181, miR-125b,^159^ and miR-29b^160^ could be regarded as potential diagnostic biomarkers, and in the future may become therapeutic targets for the addiction to alcohol, cocaine, heroin, methamphetamine, nicotine, cannabidiol, or opiates. Interestingly, the levels of miR-16-5p, miR-129-5p, miR-363-3p, and miR-92a-3p from EVs in patients with heroin dependence and methamphetamine dependence showed a significantly negative correlation with the symptoms of anxiety,^161^ indicating that EV-related miRNAs may serve as overlapping pathogenesis of these two disorders. Moreover, a recent study focusing on acute and protracted withdrawal showed that the alterations in cytokine level and imbalance of Th1/Th2/Th17 in patients addicted to heroin corresponded with abnormalities in EV-related lncRNA/mRNA expression,^162^ which provided a novel explanation for the pathological mechanism of heroin addiction.

EVs also showed the potential of therapeutic use for SA. It has been found that intranasal administration of EVs with miR-124 alleviated cocaine-mediated microglial activation,^163^ while astrocyte-derived EVs encapsulating siRNA restored phagocytic activity of microglia and restrained morphine addiction.^164^ These studies shed light on the feasibility of EVs as a therapeutic option of SA.

### Sleep disorder

SD, associated with various factors such as stress, endocrine dysfunction, and drugs, is constantly associated with neurodegenerative diseases and affective disorders, with overlapping molecular mechanisms such as dysregulation of BDNF.^125^, ^165^, ^166^ Studies correlating EVs and sleep mainly focused on secondary sleep apnea, but herein we only discuss SD from the psychiatric perspective. EVs have been found to be involved in the pathogenesis of promoting Aβ formation, transferring tau protein, mediating neuroinflammation, and increasing BBB permeability in elderly patients with SD.^167^ A series of pre- and clinical studies have shown that some EV-related miRNAs, including miR-188-5p^168^ and miR-155,^169^ may be responsible for the pathophysiological process of SD, but none showed reliable specificity.

### Post-traumatic stress disorder

PTSD is a severe mental disorder caused by trauma and may affect neurodevelopment that result in structural and functional abnormalities in brain.^170^ Significant alternations of EV-related miRNAs in veterans with PTSD were associated with immune disturbance, including immune response and pro-inflammatory cytokines secretion.^171^ Therefore, these miRNAs may serve as candidates for monitoring the inflammatory status in PTSD individuals.^171^ Additionally, veterans with PTSD showed alterations in plasma concentration of miR-203a-3p and miR-339-5p,^172^ which would interact with genes involved in the pathogenesis of PTSD and other comorbidity conditions such as cardiovascular diseases, inflammatory reaction, and neurotransmitter system,^173^, ^174^ suggesting these diseases may share similar pathophysiological mechanisms. Although growing number of emerging studies focusing on EV-related miRNAs and other biomarkers of PTSD, most findings still require further clinical validations.

## Limitations

Research of EVs in mental disorders is still in its infant stage. Currently, most studies focus on improving the detecting techniques, identifying new EV candidates and validating their specificity in large sample size. In general, no clear conclusions have ever been drawn regarding the mechanisms of EVs underlying the pathogenesis of mental disorders.

One major limitation lies in the identification of tissue-specific EVs by liquid biopsy. In the CNS, EVs may originate from various types of cells including neurons, endothelial cells, glia, as well as peripheral immune cells migrating into the brain, or simply derive from the peripheral circulation.^19^, ^20^, ^34^, ^175^ This accounts for the diversity in the components of EVs, making it difficult to identify the specific candidates for certain mental disorder. To overcome this difficulty, brain-derived EVs such as neuron- or -glia-specific EVs, or brain region-specific EVs with specific biomarkers are being developed.

Another limitation is that few EV-based biomarkers are disease-specific and may be identified across different diseases. Also, the exact function of these EV cargoes is unclear. For example, miR-132 was observed to be dysregulated in patients with different mental disorders, including schizophrenia, MDD, and BD,^68^ and was known to participate in maintaining the integrity of brain vessels.^66^ However, whether miR-132 contributes to the neuropathology of these mental disorders via damaging the brain vessels remains unknown. Controversial findings call for special concerns. For example, the miR-142-3p was increased in two BD cohorts from USA and China respectively, while was decreased in a cohort from Turkey.^86^, ^87^ This inconsistence may generate from differences in ethnicity, size of cohorts or measurement methods. This phenomenon reminds researchers to establish standardized study design in EVs studies. Therefore, great endeavor is needed to clarify the molecular mechanisms of EVs and their cargoes in the CNS, which may further help to understand the role of EVs in mental disorders.

## Future directions

Precise diagnosis and individualized treatment are two major directions for applying EVs in clinical psychiatry. Great challenge exists in the clinical practice of psychiatry, and most importantly, diagnosis of mental disorders is almost dependent on self-report symptoms and physicians' experience. Not a single experimental indicator has specificity for any mental disorder. Previous studies have demonstrated the diagnostic potential for EVs and their cargoes.^3^ Furthermore, EVs with neuronal origin biomarkers provide novel, convenient, and safe approaches for exploring and identifying changes in the CNS. For example, neuron- and astrocyte-derived EVs in the peripheral blood and the encapsulated proteins including auto-lysosomal proteins, synaptic proteins, etc. were suggested as reliable biomarkers for early Alzheimer's disease (AD) diagnosis and even accurate predictors for the development of AD up to 10 years before its clinical onset.^176^-^178^ However, there are still some issues that need to be considered. Firstly, the reliability of EVs as biomarkers for mental disorders should be verified, since the contents of EVs and their cargo in the peripheral blood are influenced by various factors including the interactions among different organ-originated EVs and the number of EVs crossing the BBB. The peripheral level of neuron-derived EVs and their cargo may not be sensitive enough to reflect the slight changes in the CNS, and in the early stage of many mental disorders, the pathobiological brain changes does not mean simultaneous change in EVs.^178^ The inconsistency of experimental results should also be noted. Possible confounding factors include the process for extracting EVs, number of repeated freezing and thawing, antibody sensitivity, quantification methods, and contamination of blood on the extracted CSF.^178^ Unfortunately, no standardized detection process has been widely used in different studies. Moreover, new evidence has raised question on the authenticity of L1CAM, a surface marker for neural EVs.^179^ Indeed, except for plasma, the antibody of L1CAM is not necessarily neuron-specific but can originate from peripheral tissues.^180^ Therefore, it is necessary to identify more specific protein markers originating from the brain or even certain cell types. Above all, although EVs have shown the potential to be developed as brand-new biomarkers for clinical diagnosis, differential diagnosis, and even rating the severity of symptoms of mental disorders, it is still in its infancy and needs more explorations.

In terms of gene therapy, EVs show advantages in targeting ability, therapeutic effect, high safety, and low immune response compared with traditional drug delivery systems including viruses, liposomes, and polyethyleneimine nanoparticles.^181^ EVs are considered convenient and safe drug-delivering tools for treating mental disorders, because of its ability of crossing the BBB and being navigated to the target cells or receptors after being engineered.^42^, ^182^ Indeed, pre-clinical and clinical studies on the treatment efficacy of EVs have provided preliminary evidence on its well tolerance and favorable response without severe toxic reaction.^183^, ^184^ Surface ligands of EVs enable the development of receptor-mediated cellular communication, and specific ligands can be enriched by engineering manipulation, according to the need of inhibitory or excitatory effects on the downstream pathways.^3^ EVs can act as carriers of nucleic acid fragments for the treatment of mental disorders, since miRNAs encapsulated in the EVs are protected from degradation by blood-derived ribonucleases,^185^ thus helping keep their integrity when transported to distant tissues.^3^ EV-siRNAs have been used to treat neurodegenerative diseases in mice,^14^ while EV-derived miR-124a could enhance the expression of excitatory amino acid transporter 2 on the surface of astrocytes, which modulates synapse activity and alleviates neuronal apoptosis.^186^ EVs derived from human mesenchymal stem cells are neuroprotective by inhibiting neuronal cell apoptosis, promoting nerve repair and regeneration, as well as restoring bioenergy following energy consumption induced by glutamate excitotoxicity.^187^ EVs and their cargo such as cystatin C also assist in the process of degrading Aβ,^188^, ^189^ which helps delay the progression of neurodegeneration. Given the complex origin of EVs, it is necessary to combine EVs biomarkers with other techniques such as MRI imaging.^190^

Notably, engineered EVs were not only a novel anticancer treatment via small non-coding RNAs , but also showed potential applications in the management of morphine addiction when modified with rabies viral glycoprotein or curcumin.^191^-^193^ When modified with specific molecules such as brain homing peptide and neuropilin-1-targeted peptide, EVs can not only have the ability of targeted delivery but also obtain imaging features *in vivo*.^194^, ^195^ Based on those techniques, similar pre-clinical studies have been carried out in neuropsychiatric diseases such as ASD,^145^ and traumatic brain injury (TBI).^196^

Furthermore, since the complicated interactions in the extracellular micro-environment can hardly be reconstructed *in vitro*, and cultured cells cannot reflect the progression or fluctuation of mental disorders, novel strategies and more advanced techniques for culturing EVs *in vitro* and establishing a vivid environment for exploring the biological functions of EVs are urgently needed. One of the advanced techniques is the brain chip, a minute co-cultured system containing human neurons, BMECs, and glia under a biomimetic condition, that can be employed as an *in vitro* model for investigation of mental disorders.^197^ In addition, with the help of human induced pluripotent stem cells, the brain‐derived EVs can be further used to explore the alterations in the cellular compositions and the specific stage of mental disorders. For example, a combination of 16 new marker candidates of brain-derived EVs and neural cell‐type‐specific EV proteins was recently detected for AD, which helps to expand our knowledge of molecular mechanisms of neurodegeneration.^198^ Another promising method is the brain organoid, which helps to observe changes in brain phenotypes and provides new tools for developing new therapeutic strategies free from brain tissues of animal models or patients.^199^ However, the feasibility of these methods remains to be validated, and a prospective design with early-stage mental disorder would be desirable to detect the objective biomarkers for timely diagnosis and treatment in the future.

## Conclusion

With the rapid development of the EVs field, increasing interest has arisen regarding its potential role in the neuropathogenesis of mental disorders, such as neuroinflammation and dysfunction of the BBB. Cargoes in EVs derived from different cell types reflect the dynamic status of diseases and thus can act as monitor and reveal pathophysiological mechanisms. Therefore, EVs seem to be ideal biomarkers of mental status and can promote non-invasive, precise diagnosis and treatment for neuropsychiatric diseases. In the case of diseases, EVs can mediate neuroinflammation and increase the BBB permeability. While under physiological conditions, EVs are necessary mediators of cellular communication between neurons and glia. Changes in EVs from CSF and peripheral biofluids such as serum/plasm, as well as their cargoes, have been detected in patients with various mental disorders such as schizophrenia, MDD, BD, and ASD. Therefore, EVs and their cargoes may facilitate the early diagnosis and evaluation of mental disorders, thereby improving the disease prognosis. However, challenges are also great. Few EVs is disease-specific and their exact role in the pathogenesis of mental disorders is largely unclarified. Moreover, studies with large clinical cohorts were also warranted to verify previous findings. Taken together, identifying the functions of EVs with high histological or pathophysiological specificity will help shed light on the pathophysiological mechanisms of mental disorders and drive the development of psychiatry.

## Figures and Tables

**Table 1:** Typical pre-clinical and clinical studies on emerging EV-derived biomarkers for ASD, SA, SD, and PTSD*

Year	Disease	Subjects	Sample source	Analysis methods	Results / Biomarkers	Ref.
2018	ASD	TG: BTBR T+tf/J mice, C57BL mice (MSC-derived EVs via intranasal administration) CG: BTBR T+tf/J mice, C57BL mice (saline via intranasal administration)	-	NanoSight analysis, NanoSight technology, western blot, etc.	Intranasal administration of MSC derived EVs increased male-to-male social interaction and reduced repetitive behaviors.	145
2019		NSC cell lines obtained from single IPSC clones from patients with ASD	Supernatants of spun media	qRT-PCR, TEM, western blot, northern blot, etc.	miR-1290	200
2020		TG: Male Shank3B C57BL/6J mice (20 μl MSC derived EVs, 107 particles/μl via intranasal administration) CG: Male wild-type littermate mice (placebo via intranasal administration)	Brain homogenates	qRT-PCR, etc.	Intranasal treatment with MSC-derived EVs improved the core ASD-like deficits of the mouse model of autism.	150
2018	ASD	TG: children with ASD (n = 20) CG: healthy children (n = 8)	Serum	Western blot, TEM, ELISA, etc.	mtDNA, IL-1β	146
2021		TG: children with ASD (n=100) CG: healthy controls (n=60)		Immunosorbent assay, TEM, NTA, western blot, qRT-PCR, etc.	EDNRA, SLC17A6, HTR3A, OSTC, TMEM165, PC-5p-139289_26, and miR-193a-5p	201
2021		TG: children with ASD (n=14); women with healthy pregnancies (n=30) CG: healthy children (n=14); women with spontaneous preterm birth (n=38), preeclampsia (n=19), gestational diabetes mellitus (n=34)	Plasma	TEM, NTA, western blot, qRT-PCR, etc.	lncRNAs of *SLC18A2*, *SYT9*, *STX8*, and *SYT15* genes mRNA of *SLC18A2*, *SYT15*, *STX8*, *SV2C*, *SYT9*, and* SYP* genes	202
2010	SA	TG: Male ICR mice with morphine pellets implanted (1× 75 mg of morphine base/pellet/mouse) CG: Male ICR mice with placebo pellets implanted (1× 75 mg of placebo base/pellet/mouse)	Brain homogenates	Western blot, qRT-PCR, polysome analysis, etc.	miR-15b, miR-181, miR-125b	159
2012		TG: Indian strain rhesus macaques with simian immunodeficiency virus affected and Morphine injected CG: Indian strain rhesus macaques with simian immunodeficiency virus affected and saline-injected		Western blot, virtual northern blot, electron microscopy, luciferase activity assays, qRT-PCR, etc.	miR-29b	160
2016		TG: Adult male C57BL/6N mice with chronic cocaine intraperitoneal injection (20 mg/kg) CG: Adult male C57BL/6N mice with placebo intraperitoneal injection (20 mg/kg)		Percoll gradient centrifugation, flow cytometry analysis, etc.	miR-124	155
2019		TG: 8-week-old male ApoE-/- C57BL/6 mice (high-fat diet), 8-week-old male ApoE-/- C57BL/6 mice (high-fat diet + nicotine) CG: 8-week-old male ApoE-/- C57BL/6 mice (normal chow diet)	vascular smooth muscle and macrophage cells	TEM, qRT-PCR, etc.	miR-21-3p	158
		Neurons and astrocytes derived from C57/BL6 wild-type, TLR4-Knock-out, and transgenic β actin DsRed mice	Supernatants of spun media	Flow cytometry analysis, NTA, TEM, etc.	TLR4, miR-146a, miR-182	154
		LN18 (chemo-resistant GBM cell line, grade IV glioblastoma derived from a male patient with a right temporal lobe glioma) LN229 (chemo-sensitive GBM cell line, glioblastoma derived from a female patient with right frontal parietal-occipital glioblastoma)	Supernatants of spun media	Western blot, NTA, miRNA analysis, etc.	miR-21, miR-126	156
2020	SA	TG: heroin-dependent patients (n=42), methamphetamine-dependent patients (n=42) CG: health controls (n=42)	Serum	Microarray-based miRNA analysis, qRT-PCR, etc.	miR-9-3p	157
2021		TG: methamphetamine-dependent patients (n=10), heroin-dependent patients (n=10) CG: healthy controls (n=10)	Plasma	Western blot, TEM, NTA, qRT-PCR, etc.	miR-16-5p, miR-129-5p, miR-363-3p, miR-92a-3p	161
2019	SD	TG: adult female mice with miR-155 knockout CG: female wild-type littermate control mice	Serum	Enzyme immunoassay, etc.	miR-155	169
2017	SD	TG: narcolepsy patients (n=20) CG: healthy controls (n=17)	Peripheral blood	qRT-PCR, miRNA microarray analysis, etc.	miR-188-5p	168
2020	PTSD	TG: FKBP5 KO mice (male and female) CG: littermate wild type (male and female)	Dissected medial prefrontal cortex	miRNA extraction technology, etc.	Circulating EV-related miRNAs showed an altered expression in FKBP5 knockout mice	203
2014	PTSD	TG: PTSD patients (n=30) CG: healthy controls (n=42)	Peripheral blood	Flow cytometric analysis, miRNA array assays, qRT-PCR, etc.	miR-125a	171
2019		TG: male Iraq and Afghanistan combat veterans with PTSD (n=12) CG: male Iraq and Afghanistan combat veterans without PTSD (n=12)	Plasma EV plasma EV-depleted plasma	qRT-PCR, small RNA sequencing data analysis, etc.	miR-203a-3p, miR-339-5p	172
		TG: military personnel with mTBI (n=42) CG: healthy controls (n=22)	Plasma	Electro-chemiluminescent immunoassays, NTA, digital array technology, etc.	Exosomal IL-10 levels were related to PTSD symptoms in military personnel with mTBI.	204
2020		TG: patients with PTSD (n=48) CG: healthy controls (n=47)	Serum	Structural and perfusion magnetic resonance, arterial spin labeling, etc.	miRNA expression levels of composite markers may be associated with PTSD symptom severity	203
2021		Patients with TBI and/or PTSD (n=144)	Plasma	miRNA profiling analysis, ELISA, etc.	Plasma neurofilament light chain, miR-139-5p	205

* Yellow: pre-clinical studie; blue: clinical studies. All studies are listed in a chronological order.ASD: autism spectrum disorder; SA: substance abuse; SD: Sleep disturbance; PTSD: post-traumatic stress disorder; mTBI: mild traumatic brain injury; TG: test group; CG: control group; PCA: principal component analysis; MSC: mesenchymal stem cell; TEM: transmission electron microscope; ELISA: enzyme-linked immunosorbent assay; mtDNA: mitochondrial DNA; miRNA/miR: microRNA; lncRNA: long non-coding RNA; NTA: nanoparticle tracking analysis; qRT-PCR: quantitative real-time polymerase chain reaction; EV: extracellular vesicles; Ref: Reference.

## Abbreviations

EVs: extracellular vesicles
BBB: blood-brain barrier
BMECs: brain microvascular endothelial cells
miRNA /miR: microRNA
lncRNA: long non-coding RNA
mtDNA: mitochondrial DNA
CNS: central nervous system
CSF: cerebrospinal fluid
BD: bipolar disorder
MDD: major depressive disorder
ASD: autism spectrum disorder
SA: substance abuse
SD: sleep disorder
PTSD: post-traumatic stress disorder
TBI: traumatic brain injury
AD: Alzheimer's disease
L1CAM: L1 cell adhesion molecule
ALIX: apoptosis-linked gene 2-interacting protein X
TSG101: tumor susceptibility gene 101
ARF6: adenosine diphosphate ribosylation factor 6
MHC: major histocompatibility complex
Aβ: amyloid-beta
BDNF: brain-derived neurotrophic factor
MSC: mesenchymal stem cell
TEM: transmission electron microscope
NTA: nanoparticle tracking analysis
GABA: gamma-aminobutyric acid
GBA: gut-brain axis
TG: test group
CG: control group
PCA: principal component analysis
ELISA: enzyme-linked immunosorbent assay
qRT-PCR: quantitative real-time polymerase chain reaction
ACC: anterior cingulate cortex
Ref: Reference
